# Changes in cerebellar output abnormally modulate cortical myoclonus sensorimotor hyperexcitability

**DOI:** 10.1093/brain/awad384

**Published:** 2023-11-13

**Authors:** Anna Latorre, Lorenzo Rocchi, Giulia Paparella, Nicoletta Manzo, Kailash P Bhatia, John C Rothwell

**Affiliations:** Department of Clinical and Movement Neurosciences, UCL Queen Square Institute of Neurology, University College London, London WC1N 3BG, UK; Department of Clinical and Movement Neurosciences, UCL Queen Square Institute of Neurology, University College London, London WC1N 3BG, UK; Department of Medical Sciences and Public Health, University of Cagliari, Cagliari 09042, Italy; Department of Neurology, IRCCS Neuromed, Pozzilli, IS 86077, Italy; Department of Human Neurosciences, Sapienza University of Rome, Rome 00185, Italy; Department of Neurology, IRCCS San Camillo Hospital, Venice 30126, Italy; Department of Clinical and Movement Neurosciences, UCL Queen Square Institute of Neurology, University College London, London WC1N 3BG, UK; Department of Clinical and Movement Neurosciences, UCL Queen Square Institute of Neurology, University College London, London WC1N 3BG, UK

**Keywords:** cortical myoclonus, cerebellum, tDCS, hyperexcitability, plasticity

## Abstract

Cortical myoclonus is produced by abnormal neuronal discharges within the sensorimotor cortex, as demonstrated by electrophysiology. Our hypothesis is that the loss of cerebellar inhibitory control over the motor cortex, via cerebello-thalamo-cortical connections, could induce the increased sensorimotor cortical excitability that eventually causes cortical myoclonus. To explore this hypothesis, in the present study we applied anodal transcranial direct current stimulation over the cerebellum of patients affected by cortical myoclonus and healthy controls and assessed its effect on sensorimotor cortex excitability. We expected that anodal cerebellar transcranial direct current stimulation would increase the inhibitory cerebellar drive to the motor cortex and therefore reduce the sensorimotor cortex hyperexcitability observed in cortical myoclonus.

Ten patients affected by cortical myoclonus of various aetiology and 10 aged-matched healthy control subjects were included in the study. All participants underwent somatosensory evoked potentials, long-latency reflexes and short-interval intracortical inhibition recording at baseline and immediately after 20 min session of cerebellar anodal transcranial direct current stimulation. In patients, myoclonus was recorded by the means of surface EMG before and after the cerebellar stimulation.

Anodal cerebellar transcranial direct current stimulation did not change the above variables in healthy controls, while it significantly increased the amplitude of somatosensory evoked potential cortical components, long-latency reflexes and decreased short-interval intracortical inhibition in patients; alongside, a trend towards worsening of the myoclonus after the cerebellar stimulation was observed. Interestingly, when dividing patients in those with and without giant somatosensory evoked potentials, the increment of the somatosensory evoked potential cortical components was observed mainly in those with giant potentials.

Our data showed that anodal cerebellar transcranial direct current stimulation facilitates—and does not inhibit—sensorimotor cortex excitability in cortical myoclonus syndromes. This paradoxical response might be due to an abnormal homeostatic plasticity within the sensorimotor cortex, driven by dysfunctional cerebello-thalamo-cortical input to the motor cortex. We suggest that the cerebellum is implicated in the pathophysiology of cortical myoclonus and that these results could open the way to new forms of treatment or treatment targets.

## Teaser

Latorre *et al.* provide evidence that dysfunctional cerebellar input to the motor cortex contributes to the sensorimotor hyperexcitability observed in cortical myoclonus. The findings clarify the pathophysiology of cortical myoclonus and suggest new treatment targets.

## Introduction

Cortical myoclonus is a jerky involuntary movement produced either by abrupt muscle contraction (positive myoclonus) or sudden cessation of ongoing muscular activity (negative myoclonus).^1^ Cortical myoclonus is produced by abnormal neuronal discharges within the sensorimotor cortex, as demonstrated by electrophysiology.^2-4^ The distinctive electrophysiological markers that differentiate cortical from subcortical myoclonus include EEG discharges time-locked to individual myoclonic jerks detected with jerk-locked back averaging (JLBA), giant somatosensory evoked potentials (SEP) and enhanced long-latency reflex type I (LLR-I), commonly referred to as C-reflex. These features suggest that hyperexcitability of the sensorimotor cortex is the pathophysiological hallmark of cortical myoclonus, as supported also by transcranial magnetic stimulation (TMS) studies. Reduced short-interval intracortical inhibition (SICI) is a common finding in cortical myoclonus syndromes,^5,6^ but reduced interhemispheric inhibition and increased intracortical facilitation have also been found,^5,7-9^ strengthening the notion of enhanced cortical excitability and reduced cortical inhibition in cortical myoclonus. However, whether the sensorimotor cortex is the site of primary abnormality or its hyperexcitability is due to abnormal input into this cortical area, is still not known.

Cortical myoclonus manifestations are diverse and form a continuum from reflex myoclonus to myoclonic epilepsy, including spontaneous myoclonus and cortical tremor.^10,11^ These motor phenomena are all ultimately caused by a sudden and brief activation of the corticospinal tract neurons, but the mechanisms underlying the discrete clinical entities within this spectrum (from localized reflex jerks to widespread activation of the sensorimotor cortex and beyond) are complex and comprise a spatially limited cortical focus of increased excitability, sustained rhythmic activity of local circuits, suppression of inhibitory circuits and spread of the excitatory bursts to wide areas of the cortex.^10^ In a recent article, we speculated on the possible mechanisms that generate each element of the spectrum, providing evidence for the cerebellum as a possible common pathophysiological denominator.^10^ The involvement of the cerebellum in spontaneous/reflex cortical myoclonus is supported by several clinical, pathological and electrophysiological evidence.^12-15^

Our hypothesis is that the loss of cerebellar inhibitory control over the motor cortex, via cerebello-thalamo-cortical (CTC) connections, could be the basis of increased sensorimotor cortical excitability that eventually causes cortical myoclonus^10^; however, direct evidence for this is still lacking.

One way to explore this hypothesis is by modulating cerebellar output and assessing its effect on sensorimotor cortex excitability. Transcranial direct current stimulation (tDCS), a non-invasive brain stimulation technique consisting of direct current delivered transcutaneously through surface electrodes,^16,17^ is a powerful tool able to modulate cerebellar excitability. TDCS effect is produced by creating a potential difference between two electrodes, which induces a subthreshold shift of neuronal resting membrane potentials towards depolarization or hyperpolarization, depending on the current flow direction relative to axonal orientation.^18^ The general rule is that anodal tDCS increases neuronal excitability, whereas cathodal tDCS exerts the opposite effect.^19^ Although the tDCS effect is not always predictable, as it also depends on the orientation of the underlying neurons and the sensitivity of their compartments to exogenous current,^20^ previous studies have shown that cerebellar tDCS can modulate, in a polarity-specific fashion, the excitability of cerebellar cortical neurons and, consequently, the output from cerebellar nuclei to the motor cortex^17,21,22^; more specifically, it has been observed that anodal tDCS increases the inhibitory action of the cerebellum to the motor cortex.^23-26^

The aim of this study was to explore whether the sensorimotor cortex hyperexcitability observed in cortical myoclonus is due to decreased cerebellar output to this area. To do so, we applied anodal tDCS over the cerebellum of patients affected by spontaneous/reflex cortical myoclonus, with the intent to increase cerebellar cortical excitability, and assess its effect on the abnormal sensorimotor cortex excitability detected in these patients. A possible effect of the stimulation on the myoclonic jerks was also evaluated. We hypothesized that anodal cerebellar tDCS (ac-tDCS) would increase the inhibitory cerebellar drive to the motor cortex, reduce the sensorimotor cortex hyperexcitability related to cortical myoclonus and therefore improve myoclonus.

## Materials and methods

### Subjects

Ten patients affected by cortical myoclonus (eight female, age 44.8 ± 19.8) of various aetiology and 10 age-matched (five female, age 43 ± 12.4) healthy control subjects were included in the study. The diagnosis of cortical myoclonus was supported by the clinical features (body distribution, combination of positive and negative myoclonus, stimulus sensitivity) and the aetiology of the syndrome,^27^ and confirmed by the presence of at least one of the following criteria: giant SEP, positive JLBA and presence of C-reflex.^2,3^ Other electrophysiological features that were considered supportive of the cortical origin of the jerks were EMG burst duration <50 ms, cranial-caudal progression of the jerks, and the presence of both positive and negative myoclonus.^2^ Demographic and clinical data were collected. Cortical myoclonus clinical features were evaluated by a movement disorders expert and cortical myoclonus severity assessed with the Unified Myoclonus Rating Scale (UMRS).

Participants underwent surface EMG recording of myoclonus (in patients), SEP, LLR and TMS recording at baseline (T0) and immediately after (T1) 20 min session of ac-tDCS applied over the cerebellum, as detailed below. The UMRS was reassessed at T1. All the tests were performed in one session, with patients OFF cortical myoclonus medications (Table 1) for at least 12–24 h. All patients underwent a brain MRI scan within 6 months prior to the study as part of their diagnostic work-up or follow-up. Healthy controls had no history of neuropsychiatric disorders and were not taking drugs active at the CNS level at the time of the experiments. Patients were not informed about any possible change (improvement/worsening) of the myoclonus due to the stimulation, to reduce the possibility of placebo effect. All procedures were carried out with the adequate understanding and written informed consent of the subjects prior to the experiments. The experiments were conducted in accordance with the Declaration of Helsinki and to international safety guidelines. Formal approval to conduct the experiments was obtained from the local ethics committee.

**Table 1:** Summary of the clinical features

General clinical features						Myoclonus clinical features					
Subject	Age (y)	Diagnosis	DD (y)	Treatment	UMRS	Distal	F	M/G	Rest	Act	Stim sens
1	27	AMRF (MYC-*SCARB2*)	6	CLZ 1 mg	85	+	−	+	+	+	−
2	70	CBS	8	L-DOPA 300 mg LVT 500 mg VPA 300 mg CLZ 0.5 mg	114	+	−	+	+	+	+
3	45	EPC	15	−	36	+	+	−	−	+	−
4	57	FCMTE	30	LVT 500 mg	135	+	−	+		+	+
5	73	Coeliac disease	20	LVT 1000 mg VPA 400 mg	138	+	−	+	+	+	+
6	25	BHC (*PDE10A*)	17	−	30	+	+	−	−	+	+
7	34	PLAN	1.5	L-DOPA 400 mg	93	+	+	−	−	+	+
8	33	Cerebellar hypoplasia	11	CLZ 1 mg VPA 600 mg	44	+	+	−	+	+	+
9	20	FCMTE	10	CLZ 1 mg VPA 800 mg LVT 1000 mg	90	+	+	−	−	+	+
10	64	FCMTE	35	VPA 800 mg	120	+	−	+	+	+	+
AV ± SD	44.8 ± 19.8		15.4 ± 10.6		88.5 ± 40.1						

Act = action; AMRF = action myoclonus renal failure syndrome; AV = average; BHC = benign hereditary chorea; CBS = cortico-basal syndrome; CLZ = clonazepam; DD = disease duration (in years); EPC = epilepsia partialis continua; F = focal; FCMTE = familial cortical tremor myoclonus epilepsy; LVT = levetiracetam; M/G = multifocal/generalized; PLAN = PLA2G6-associated neurodegeneration; SD = standard deviation; Stim sens = stimulus sensitive; VPA = valproic acid; UMRS = Unified Myoclonus Rating Scale; + = present; − = absent.

### Myoclonus recording

The myoclonus was recorded by means of surface EMG from the most affected muscle, based on visual inspection. As all patients had upper limb distal myoclonus, EMG was recorded from an arm or hand muscle [mainly the extensor carpi radialis, flexor carpi radialis or the abductor pollicis brevis (APB) muscle]. EMG activity was recorded using Ag/AgCl electrodes placed in a bipolar fashion on the belly of the selected muscle for ∼60 s, with acquisition parameters similar to those used for motor evoked potentials (MEP). The root mean square (RMS) of the EMG signal was calculated and values were used for statistical analyses.

### Recording and analysis of somatosensory evoked potentials

SEP were recorded from two Ag/AgCl electrodes placed according to the 10–20 international EEG system at CP3/4 (active) and Fz (reference electrode). Skin impedances were kept below 5 kΩ. To get SEP, the median nerve (of the most affected side in patients and right side in healthy controls) was stimulated with a constant-current stimulator (DS7A, Digitimer Ltd). The anode was placed on the wrist crease and the cathode 2 cm proximal. Monophasic square wave pulses of 200 µs duration were delivered at 250% of the somatosensory threshold at a frequency of 3 Hz ± 10%, and 500 trials were collected in each block.^28,29^ Signal was recorded from −20 to 100 ms around the pulse, digitized with a 5 KHz sampling frequency and band-pass filtered (3 Hz–2 KHz).^28^ Peak-to-peak amplitude of N20-P25 and P25-N33 components was measured. N20, P25 and N33 latency were measured. SEP were considered giant when the amplitudes of the N20-P25 and P25-N33 components both exceeded normal values by 3 standard deviations (SD), obtained in a sample of 20 age-matched healthy subjects.^30-32^ According to this criterion, patients were divided in those with and without giant SEP. The percentage increase of SEP amplitude, for each SEP component, was calculated as: [(SEP amplitude at T1 − SEP amplitude at T0) / SEP amplitude at T0] × 100.

### Recording and analysis of long-latency reflexes

LLRs were obtained by following current guidelines.^33^ Median nerve stimulation was performed as for SEP, but with an intensity able to evoke a compound muscle action potential from the APB muscle at rest of ∼100–200 µV. EMG was recorded from the same muscle, with acquisition parameters similar to those used for MEP (see later), at rest in both patients and healthy controls and at 30% of maximum voluntary contraction (MCV) in controls only. One block of 500 trials was recorded. Peak-to-peak amplitude of LLR-I (35–46 ms), LLR-II (45–58 ms) and LLR-III (>68 ms)^34^ were measured when present.

### Transcranial magnetic stimulation and EMG recording and analysis

EMG activity was recorded using Ag/AgCl electrodes placed over the first dorsal interosseous (FDI) muscle of the most affected hand in patients and the right hand in healthy controls, in a belly-tendon fashion. EMG signal was bandpass filtered (5 Hz–2 kHz) and digitized at 5 kHz. Data were stored in a laboratory computer for on-line visual display and further off-line analysis (Signal software, Cambridge Electronic Design, Cambridge, UK). TMS was performed using a Magstim 200 monophasic stimulator with a 70 mm figure-of-eight coil (Magstim Company Limited). First, the motor hotspot was found, defined as the site within the primary motor cortex (M1) where the largest MEP in the contralateral FDI could be obtained. Then, we measured the active motor threshold (AMT) and the intensity able to elicit MEP of ∼1 mV (1 mV-int) amplitude from the FDI muscle, which was later used for test stimuli (TS). AMT was defined as the lowest intensity able to evoke a MEP of at least 200 µV in 5 out 10 consecutive trials, during a slight tonic contraction of the target muscle at ∼10% of the MCV.^35^ SICI was tested in the hemisphere contralateral to the most affected hand in patients and over the left hemisphere in healthy controls, and obtained through paired-pulse TMS, with an interstimulus interval (ISI) of 3 ms between the conditioning stimulus (CS) and TS. The TS was set at 1 mV-int, while the CS was set at 70%, 80%, 90% and 100% AMT, to obtain a recruitment curve.^29,36^ Fifteen TS and 15 pairs of a CS followed by a TS for each CS intensity were given in a pseudo-randomized order. Amplitude of MEP elicited by TS alone and by CS-TS pairs were measured peak-to-peak. SICI was calculated as the amplitude ratio between conditioned (CS-TS) and test stimuli.

### Transcranial direct current stimulation

TDCS was delivered via two 5 × 5 cm sponge electrodes soaked in saline solution. The anode was placed 3 cm lateral to the inion on the cerebellar hemisphere ipsilateral to the most affected side in patients and on the right cerebellar hemisphere in healthy controls. The cathode was positioned on the buccinator muscle, ipsilateral to the active electrode. TDCS was given for 20 min at an intensity of 2 mA.^21,37^ At the beginning of stimulation, the current was increased gradually from 0 to 2 mA over 30 s.

### Statistical analysis

Two two-way mixed ANOVA with factors ‘Group’ (patients, healthy) and ‘Time’ (T0, T1) were performed to assess the effect of ac-tDCS on the amplitude of N20-P25 and P25-N33 components of SEP, respectively, and to assess possible baseline differences between the two groups. Several dependent *t*-tests were used to evaluate the effect of ac-tDCS on SEP components latencies within each group. As the LLRs were recorded in different conditions in the two groups (at rest patients and during muscle contraction in controls), we investigated the effects of ac-tDCS on LLR amplitude in the two groups separately by means of two paired *t*-tests. A two-way mixed ANOVA with factors Group (patients, healthy) and Time (T0, T1) was performed to assess the effect of ac-tDCS on test MEP and to assess possible baseline differences between the two groups. A three-way mixed ANOVA with factors Group (patients, healthy), Time (T0, T1) and ‘Conditioning’ (70%, 80%, 90%, 100% AMT) was performed to assess the effect of ac-tDCS on SICI. Last, a paired *t*-test was performed to assess possible differences in EMG RMS values induced by ac-tDCS in patients. Correlations between variables were evaluated with the Spearman’s rank correlation coefficient. Normality of distribution was assessed with the Shapiro-Wilk test, while Greenhouse-Geisser correction was used, if necessary, to correct for non-sphericity (i.e. Mauchly’s test < 0.05). *P*-values < 0.05 were considered significant. All main effects, interactions and *post hoc* tests were Bonferroni-corrected.

## Results

All participants completed the study and reported no side effects from the cerebellar stimulation. The demographic (including age at the time of the study, diagnosis, disease duration and UMRS value) and clinical features (including myoclonus distribution and activation state) of the patients are detailed in Table 1. At baseline, the mean UMRS value was 88.5 ± 40.1 and did not differ from the post-ac-tDCS value (90 ± 43.8). Brain MRI disclosed cerebellar atrophy in Case 5 and cerebellar hypoplasia in Case 8, the other MRIs did not show any cerebellar abnormality. The electrophysiological and other relevant findings to support the diagnosis of cortical myoclonus and salient MRI results are summarized in Table 2.

**Table 2:** Summary of the electrophysiological or other diagnostic relevant findings

Subject	Giant SEP	LLR-I	JLBA	<50 ms	Cranio caudal	Pos and Neg	EEG	Others
1	−	+	+	+	+	+	N/A	-
2	+	+	+	+	+	+	N/A	Abnormal DaT scan MRI: symmetrical pattern of frontal and parietal atrophy
3	−	+	+	+	−	+	Ictal sharp activity over the left centroparietal region	
4	+	+	N/A	+	+	+	N/A	
5	−	+	+	+	+	+	N/A	MRI: volume loss of the cerebellum and supratentorial brain
6	+	+	Major EEG artefacts	+	−	−	N/A	MRI: bilateral striatal hyperintensity in T_2_-weighted
7	−	+	+	+	+	+	N/A	MRI: GP, SN and striatum iron deposition
8	+	+	N/A	+	−	+	N/A	MRI: left cerebellar hypoplasia
9	−	+	+	+	+	−	−	
10	+	+	Major EEG artefacts	+	+	+	2–3 Hz slow waves left posterior temporo-occipital region	

EMG/NCS = electromyography/nerve conduction study; GP = globus pallidus; JLBA = jerk-locked back averaging; LLR = long-latency reflex; N/A = not available; SEP = somatosensory evoked potential; SN = substantia nigra; Pos and Neg = positive and negative; + = present; − = absent.

### Somatosensory evoked potentials

**Figure 1:**
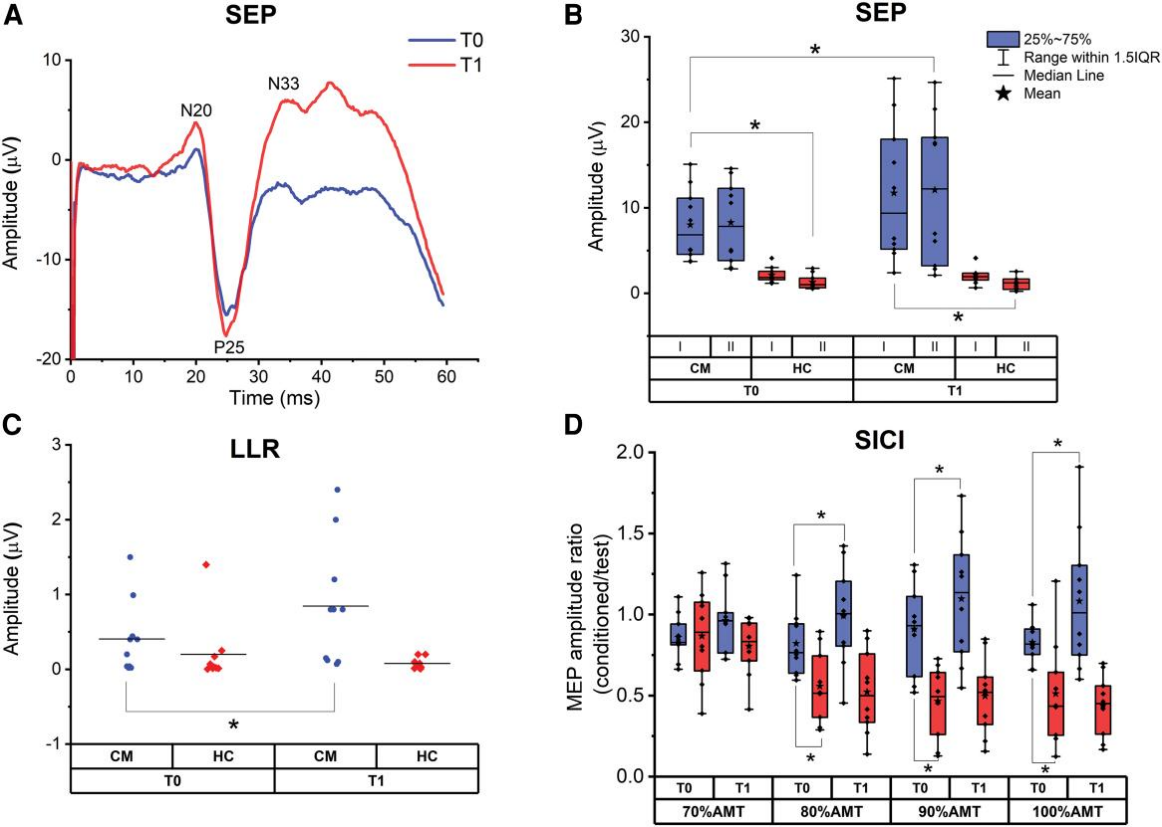
**The effect of ac-tDCS on SEP, LLR and SICI in healthy controls and patients with cortical myoclonus.** (**A**) Example of giant somatosensory evoked potentials (SEP) recorded from a patient at baseline (T0) and immediately after (T1) 20 min session of cerebellar anodal transcranial direct current stimulation (ac-tDCS). Note that SEP were considered giant when the amplitudes of the N20-P25 and P25-N33 components both exceeded normal values by 3 standard deviations (SD), obtained in a sample of 20 age-matched healthy subjects. (**B**) Changes of SEP components amplitude (I: N20-P25, II: P25-N33) after ac-tDCS (T1) in healthy controls (HC) and patients with cortical myoclonus (CM). *Statistically significant comparisons (*P* < 0.05): N20-P25 (I) and P25-N33 (II) amplitude was significantly higher in cortical myoclonus patients than in healthy controls, both at T0 and T1; N20-P25 (I) and P25-N33 (II) amplitude in cortical myoclonus was significantly higher at T1 compared to T0. (**C**) Changes of long-latency reflex type I (LLR-I) after cerebellar tDCS (T1) in healthy controls and cortical myoclonus patients. *Statistically significant comparisons (*P* < 0.05): LLR-I amplitude was significantly higher in cortical myoclonus at T1 compared to T0. (**D**) Short-interval intracortical inhibition (SICI) at different intensities of the conditioning stimulus (70%, 80%, 90% and 100% AMT), in patients with cortical myoclonus and healthy controls, at T0 and T1. *Statistically significant comparisons (*P* < 0.05): at T0, SICI was significantly less in cortical myoclonus patients compared to healthy controls at conditioning stimulus intensity of 80%, 90% and 100% AMT; SICI was significantly less (turning into facilitation) in cortical myoclonus at T1 compared to T0 at conditioning stimulus intensity of 80%, 90% and 100% AMT. The box chart legend is the same as **B**. Blue = patients with cortical myoclonus; red = healthy controls. AMT = active motor threshold; MEP = motor evoked potential.

SEPs were considered giant if N20-P25 amplitude was > 5.54 µV and P25-N33 amplitude was > 4.30 µV. According to this criteria, 5/10 patients had giant SEP (values are shown in Table 3). Ac-tDCS had no effect on the latency of SEP components (*P* values of all tests > 0.05), but significantly increased their amplitude in patients: the ANOVA on N20-P25 amplitude showed a significant main effect of Group [*F*(1,18) = 16.076, *P* < 0.001], Time [*F*(1,18) = 7.007, *P* = 0.016] and a significant Group × Time interaction [*F*(1,18) = 6.641, *P* = 0.019]. *Post hoc* comparisons showed that N20-P25 amplitude was higher in patients than in healthy controls, both at baseline (*P* < 0.001) and after ac-tDCS (*P* = 0.002). Interestingly, ac-tDCS led to significant increase in N20-P25 amplitude in patients (*P* = 0.002), while it had no significant effect in healthy controls (*P* = 0.961) (Fig. 1A and B). These effects were confirmed by the ANOVA on P25-N33 amplitude. There was a significant main effect of Group [*F*(1,18) = 18.260, *P* < 0.001], Time [*F*(1,18) = 6.227, *P* = 0.023] and a significant Group × Time interaction [*F*(1,18) = 7.565, *P* = 0.013]. *Post hoc* comparisons showed that P25-N33 amplitude was higher in patients than in healthy controls, both at baseline (*P* < 0.001) and after ac-tDCS (*P* = 0.001). Again, ac-tDCS led to a significant increase in P25-N33 amplitude in patients (*P* = 0.002), while it had no significant effect in controls (*P* = 0.859) (Fig. 1A and B).

**Table 3:** Somatosensory evoked potential amplitudes

	T0 (µV)		T1 (µV)	
	N20-P25	P25-N33	N20-P25	P25-N33
**CM**				
1	5.11	3.01	5.16	3.21
2	**10.12**	**10.57**	**15.30**	**18.23**
3	5.03	2.87	5.78	2.10
4	**15.09**	**14.11**	**22.04**	**21.55**
5	3.71	3.83	2.40	2.84
6	**11.11**	**11.43**	**25.13**	**24.66**
7	3.78	5.07	4.70	6.11
8	**8.55**	**14.60**	**12.33**	**17.61**
9	4.55	4.91	6.41	6.99
10	**13.02**	**12.27**	**18.03**	**17.44**
AV ± SD	**8.01 ± 4.15**	**8.27 ± 4.76**	**11.72 ± 8.04**	**12.07 ± 8.63**
**HC**				
1	3.01	1.33	4.12	1.34
2	1.67	1.12	1.55	1.66
3	4.12	2.55	4.13	2.55
4	1.76	1.77	1.23	1.29
5	1.34	0.55	2.35	0.65
6	1.58	0.68	2.11	0.27
7	2.24	0.65	1.78	1.13
8	1.17	0.89	0.65	0.23
9	1.92	2.93	1.62	1.66
10	2.56	0.62	2.33	0.46
AV ± SD	**2.14 ± 0.89**	**1.31 ± 0.85**	**2.19 ± 1.14**	**1.12 ± 0.74**

The values in bold indicate the giant somatosensory evoked potentials. T0 refers to measures collected at baseline. T1 refers to measures collected after 20 min session of ac-tDCS. AV = average; CM = cortical myoclonus; HC = healthy controls; SD = standard deviation.

**Figure 2:**
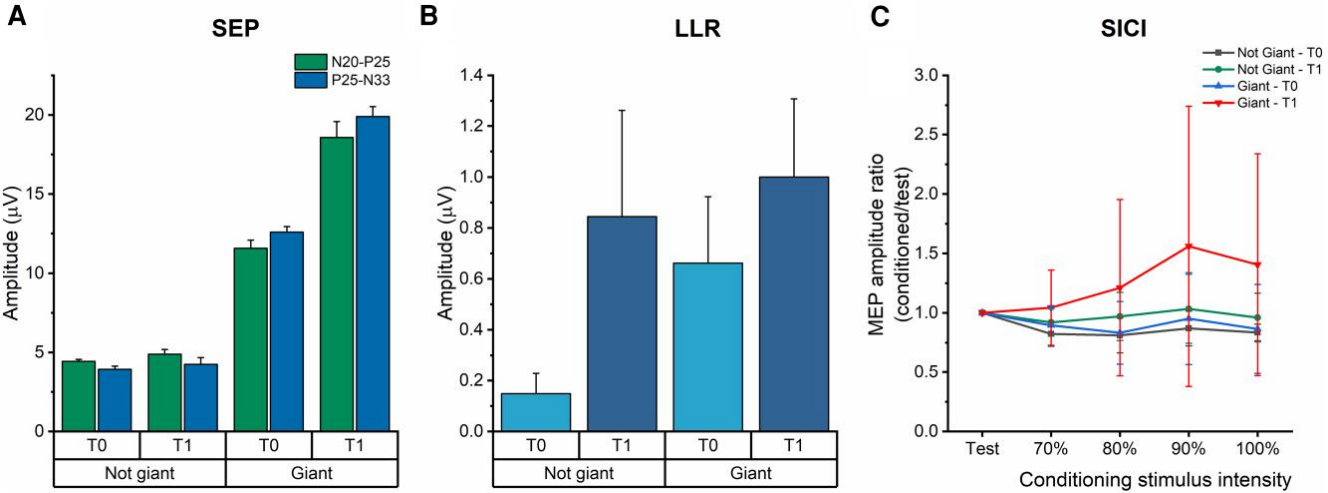
**The effect of ac-tDCS on SEP, LLR and SICI patients with cortical myoclonus, with and without giant SEP.** (**A**) Changes of somatosensory evoked potentials (SEP) components amplitude after cerebellar anodal transcranial direct current stimulation (ac-tDCS) (T1) in the two groups of patients with and without giant SEP. (**B**) Changes of long-latency reflex type I (LLR-I) after ac-tDCS (T1) in the two groups of patients with and without giant SEP. (**C**) Short-interval intracortical inhibition (SICI) at different intensities of the conditioning stimulus (70%, 80%, 90% and 100% AMT) in patients with and without giant SEP at T0 and T1. Statistical analysis was not performed due to the small number of patients for each group. AMT = active motor threshold; MEP = motor evoked potential.

Considering the two groups of patients with and without giant SEP, the increment of the N20-P25 and P25-N33 amplitude at T1 was observed mainly in those with giant SEP (Fig. 2A): the percentage change was 9.16% (N20-P25) and 3.37% (P25-N33) in the group without giant SEP, and 61.23% (N20-P25) and 60.74% (P25-N33) in those with giant SEP. This result was confirmed by the correlation analysis, which was performed by means of the Spearman’s correlation coefficient, and showed a significant positive correlation between baseline amplitude of N20-P25 and P25-N33 SEPs and changes in SEP amplitude induced by ac-tDCS (*r* = 0.685, *P* = 0.029 and *r* = 0.636, *P* = 0.048, respectively).

### Long-latency reflexes

LLR-I (C-reflex) was present in all patients at rest; Patient 1 showed both LLR-I and LLR-III and Patient 2 showed all three peaks. In healthy controls, none of the LLRs were present at rest; however, all healthy controls showed LLR-I at 30% of MCV, 5/10 had LLR-II, three of which also had LLR-III. The *t*-test on LLR-I amplitude recorded at rest in patients showed that ac-tDCS induced a significant increase in amplitude compared to baseline [*t*(10) = −4.760, *P* = 0.001]. In healthy subjects, the same analysis showed a non-significant trend towards a decrease in LLR-I amplitude recorded during contraction [*t*(10) = 1.636, *P* = 0.136] (Fig. 1C).

We also assessed LLR-I changes in the two groups of patients with and without giant SEP. Patients without giant SEP had a lower LLR-I amplitude at baseline compared to those with giant SEP; however, they had a higher increment of amplitude after ac-tDCS compared to those with giant SEP (Fig. 2B).

### Transcranial magnetic stimulation

The ANOVA on test MEP amplitude showed a non-significant main effect of Group [*F*(1,18) = 0.183, *P* = 0.674], Time [*F*(1,18) = 0.225, *P* = 0.225] and a non-significant Group × Time interaction [*F*(1,18) = 0.225, *P* = 0.641]. This means that there was no baseline difference in MEP between the two groups and that the effect of ac-tDCS was not significant, both in patients and in healthy controls. This allowed for the final analysis on SICI, performed on ratios of conditioned/unconditioned MEPs. The ANOVA showed a significant main effect of Group [*F*(1,18) = 283.039, *P* < 0.001], a non-significant effect of Time [*F*(1,18) = 1.552, *P* = 0.229], a significant main effect of ‘conditioning’ [*F*(5,90) = 7.849, *P* < 0.001]. The analysis also disclosed significant Group × Time [*F*(1,18) = 5.659, *P* = 0.029], Group × Conditioning [*F*(5,90) = 13.267, *P* < 0.001] and Time × Conditioning [*F*(5,90)= 3.730, *P* = 0.004] interactions, while the Group × Time × Conditioning interaction was not significant [*F*(5,90) = 0.878, *P* = 0.5]. *Post hoc* comparisons showed that baseline SICI was less in patients compared to healthy controls when considering a conditioning stimulus strength of 80% (*P* = 0.011), 90% (*P* = 0.001) and 100% (*P* = 0.017) AMT. Whereas ac-tDCS had no effect on SICI in healthy controls, it further decreased SICI in patients, turning it into facilitation, at 80% (*P* = 0.006), 90% (*P* = 0.026) and 100% (*P* = 0.015) AMT intensity of the conditioning pulse (Fig. 1D).

The response of SICI to ac-tDCS has been also analysed in the groups of patients with and without giant SEP. As shown in Fig. 2C, ac-tDCS decreased SICI in patients with giant SEP to a greater extent compared to those without giant SEP. As for the SEP, there was a significant positive correlation, tested by the Spearman’s correlation coefficient, between baseline SEP amplitude and the average SICI changes across all CS intensities induced by ac-tDCS (N20-P25: *r* = 0.818, *P* = 0.004; P25-N33: *r* = 0.733, *P* = 0.016).

### Myoclonus recording

**Figure 3:**
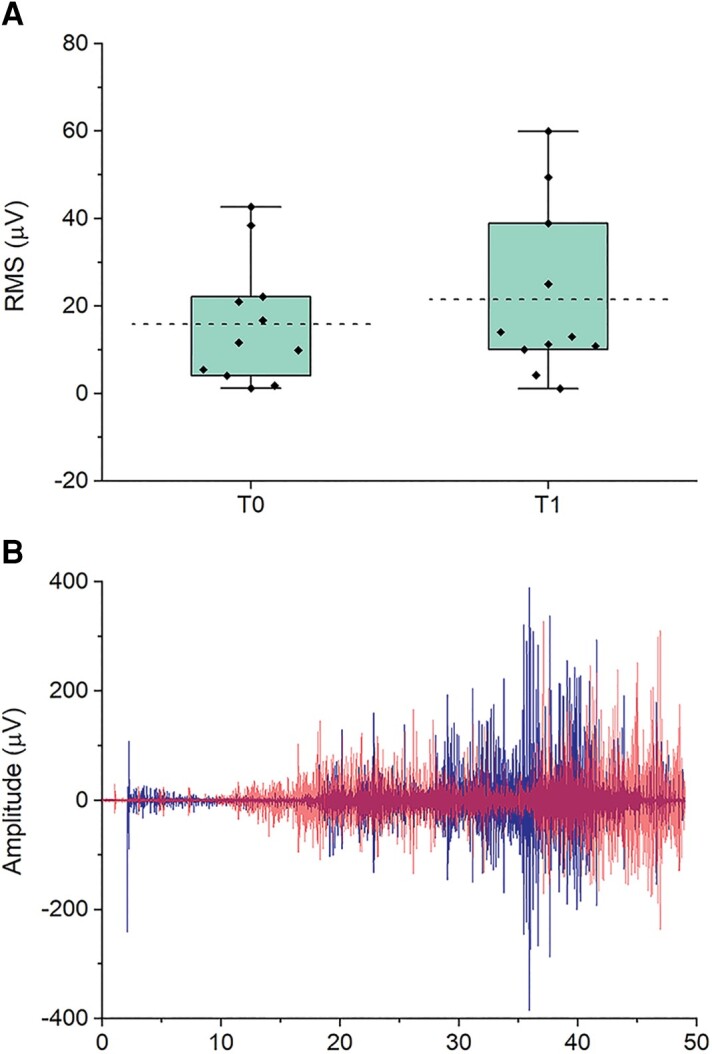
**The effect of ac-tDCS on cortical myoclonus.** (**A**) Root mean square (RMS) of the EMG myoclonic bursts at baseline (T0) and immediately after (T1) 20 min session of cerebellar anodal transcranial direct current stimulation (ac-tDCS). Boxes indicate 25th to 75th percentiles of data distribution. Whiskers include the whole data distribution. The dashed lines indicate the distribution mean. (**B**) Example of EMG myoclonic bursts in a patient at T0 (blue) and T1 (red).

The *t*-test on EMG RMS did not disclose a significant difference between T0 and T1, although there was a trend towards an increase (36%) in EMG activity after ac-tDCS [*t*(10) = −1.935, *P* = 0.085] (Fig. 3).

## Discussion

The present results show that ac-tDCS did not change SEP, LLR and SICI in healthy controls, while in patients with cortical myoclonus it significantly increased the amplitude of the SEP (both N20-P25 and P25-N33 components) and of LLR-I (C-reflex), and decreased SICI; there was also a trend towards worsening of myoclonus after ac-tDCS. These results are the opposite to our initial predictions, which had suggested that ac-tDCS should inhibit, and not facilitate, sensorimotor excitability in cortical myoclonus; nevertheless, they do support the underlying assumption that the cerebellum has an important role in the pathophysiology of cortical myoclonus.

### Sensorimotor excitability in cortical myoclonus compared to healthy controls

The cardinal pathophysiological marker of cortical myoclonus, compared also to other myoclonus subtypes, is the presence of sensorimotor hyperexcitability, that is thought to be responsible for abnormal neural discharges causing the myoclonic jerks. Sensorimotor hyperexcitability has been confirmed in our patients by the presence of increased SEP amplitude, LLR-I at rest, and reduced SICI compared to controls. The presence of giant SEP and LLR-I was expected as part of the inclusion criteria,^2,3^ while the finding of reduced SICI in cortical myoclonus is in line with other studies.^5,6^ SEP recording offers a non-invasive method for assessing the functions of the somatosensory pathways at different levels of the nervous system. N20 is generated in the area 3b of the primary somatosensory cortex (S1), while the generators of later components P25 and N33 seem to lie in area 1, which receives input from area 3b and from later arriving inputs from slower conducting afferents and more indirect pathways (such as via the cerebellum).^38^ Half of the patients showed no giant SEPs, as defined as amplitudes of the N20-P25 and P25-N33 components exceeding normal values by 3 SD. This finding is not surprising, as not all patients presumed to have cortical myoclonus show giant cortical responses,^2,39^ very likely because a diversity of (possibly related) mechanisms that can produce cortical myoclonus. It is possible that in some cases (and mostly in those with reflex cortical myoclonus) the motor output is driven by an abnormal sensory cortex activity, whereas in other cases it is not. It is normally assumed that SEP components are due to the activation of excitatory connections, but in cortical myoclonus this might not always be true. For instance, in epilepsia partialis continua, a form of cortical myoclonus, the absence/reduction of SEP P24 wave amplitude has been hypothesized to be related to an impairment of the GABAergic tonic inhibition in the sensorimotor cortex, mediated by an intra-cortical network rather than dysfunction of thalamo-cortical projections.^40^ This suggests that the mechanisms generating abnormal SEP in cortical myoclonus are complex and not necessarily related to thalamo-cortical input but possibly to other afferents.^38^

Although LLR-I have not always been reported in cortical myoclonus, it could be recorded in all our patients but not in healthy controls at rest. LLRs are long-latency hand-muscle reflexes likely mediated by transcortical pathways and LLR-I (C-reflex), which has a latency of 35–46 ms, is considered a key element for the neurophysiological diagnosis of cortical myoclonus. In the first description of the C-reflex, it was hypothesized that the neural pathway included peripheral nerve, dorsal funiculus of spinal cord, contralateral ventral posterior nucleus of thalamus, sensorimotor cortex, corticospinal tract and anterior horn cell, but this conclusion has not been experimentally confirmed^41^; however, recent evidence also suggests that cerebellum may be involved in LLR generation.^42^ Finally, SICI is a measure of motor intra-cortical inhibition likely mediated by GABAa interneurons.^43,44^ Reduced SICI is the most robust finding of motor cortical disinhibition in cortical myoclonus of different aetiologies,^5^ as also confirmed in our group of patients.

### Anodal cerebellar transcranial direct current stimulation effect in cortical myoclonus and healthy controls

In the present study, ac-tDCS in healthy controls did not modify any of the variables tested, namely SEP, LLR amplitude and SICI. The lack of effect on the SEP is consistent with a previous study of ac-tDCS in controls,^45^ and with clinical experience that cerebellar lesions do not cause evident sensory deficits. Nevertheless, the cerebellum may play a role in higher level sensory acquisition and discrimination.^46^ There are no previous data on the effect of ac-tDCS on LLR, although patient studies provide some evidence that the cerebellum modulates the gain of LLR.^47,48^ One previous study confirmed the present data showing that ac-tDCS has no effect on SICI,^22^ but another reported that ac-tDCS can reduce SICI.^49^ Different methods of SICI calculation could account for this discrepancy, with our results being in line with those of Galea and colleagues.^22^ In conclusion, our findings do not provide evidence that ac-tDCS can change sensorimotor excitability measured by SEP, LLR amplitude and SICI in healthy controls.

In contrast, in cortical myoclonus, ac-tDCS modified SEP, LLR amplitude and SICI, with the overall effect being an increase of sensorimotor excitability. Interestingly, the increment in SEP amplitude was observed only in patients with giant SEPs and, similarly, there was a greater reduction in SICI in the giant compared to the ‘normal’ SEP group. These results were confirmed by correlation analyses, although they should be interpreted with caution due to the small sample size. However, not all the changes were limited to patients with giant SEP, as there was a larger increase in amplitude of LLR-I after ac-tDCS in patients without giant SEP.

To the best of our knowledge, there are no other reports investigating the effect of cerebellar tDCS on SEP, LLR and SICI in cortical myoclonus. In a previous study, ac-tDCS was used with the intent of normalizing the increased long latency stretch reflexes (LLSR) in patients with cerebellar ataxia,^47^ caused by reduced inhibition of the cerebellar cortex on the deep cerebellar nuclei (DCN) in this condition.^50^ The study showed that the abnormal LLSRs, with a latency of 55–85 ms, were reduced in amplitude by the stimulation,^47^ but short latency stretch reflexes (SLSR), with a latency of 20–40 ms (of which the longer latency overlap with LLR-I), were unaffected. The different responses of SLSR to ac-tDCS in patients with cerebellar ataxia and of LLR-I in patients with cortical myoclonus could be due to the different pathophysiological processes underlying the two conditions, rather than be related only to the involvement of the cerebellum in these reflexes’ generation.

### Anodal cerebellar transcranial direct current stimulation facilitated sensorimotor excitability in cortical myoclonus patients

Ac-tDCS is thought to depolarize Purkinje cells and increase their inhibitory output to DCN. Logically, this should reduce the activity of excitatory CTC projections^51^ and reduce M1 excitability.^22,52^ This is consistent with the finding that cathodal stimulation (which reduces cerebellar inhibition of DCN) decreases the ability of cerebellar TMS to inhibit M1 (i.e. cerebellar-brain inhibition), while anodal tDCS does the opposite.^22-26,52^ Our hypothesis was that if ac-tDCS reduces M1 excitability in healthy controls, the same would happen in cortical myoclonus and that physiologically, it would reduce the SEP and LLR-I and increase SICI.

We can only speculate on why the results were opposite to those expected. One possibility is that the cerebellum in cortical myoclonus responds in the same way to ac-tDCS as healthy controls, and that the deficit lies upstream of cerebellum. It would indicate that in both controls and cortical myoclonus patients, ac-tDCS could depolarize Purkinje cells and lead to an increase activity at Purkinje cell-DCN synapses, which, if reinforced by an additional effect on the excitability of DCN dendrites,^53^ could cause a long-term potentiation (LTP)-like increase in the effectiveness of Purkinje-cell-DCN synapses and a long-term increase in suppression of DCN activity by ongoing Purkinje-cell discharge. The normal plastic response to tDCS in patients would be consistent with previous reports that cortical excitability in both groups is suppressed to the same extent by a different form of inducing-plasticity brain stimulation, i.e. inhibitory repetitive TMS to M1.^54-57^ This implies that the pathomechanism of myoclonus is not directly related to stimulation-dependent modulation of synaptic plasticity. Consequently, if ac-tDCS had the same effect on cerebellar output in cortical myoclonus patients and healthy controls, then one explanation of our results is that the abnormally excitable M1 in cortical myoclonus responds in the opposite way to removal of cerebellar facilitation. Effectively, M1 in cortical myoclonus would ‘compensate’ for the reduction in facilitation by further increasing its own excitability; hence, the paradoxical response would be an abnormal plastic response of motor cortex neurons to a change in cerebellar inputs.

This abnormality could be described as a failure of normal homeostatic mechanisms to maintain the correct level of cortical excitability. Homeostatic plasticity refers to mechanisms that counteract the destabilizing influence of synaptic plasticity and maintain neural activity within a physiologically meaningful range; it can be triggered by tDCS, which can be used to regulate the synaptic strength.^58^ We speculate that the ‘set point’ of excitability in cortical myoclonus is higher than normal and it is reflected in the increased excitability of M1 at baseline. Rather than depressing M1, removal of facilitation produces a homeostatic response that compensates by raising excitability still further. In support of this, it is interesting to note that only enlarged SEPs were increased in size after the cerebellar stimulation (Fig. 2), suggesting that the aberrant response could be induced only when acting on a formerly defective system. Similarly, cerebellar stimulation reduced SICI and turned it into facilitation, mainly in those patients with a giant SEP (Fig. 2).

A similar type of paradoxical response to changes in M1 excitability has been reported in a form of myoclonic epilepsy. Quadripulse transcranial magnetic stimulation (QPS), which is another method that interacts with synaptic plasticity, was applied over M1 to investigate its effect on S1 (as assessed by SEPs) in patients affected by benign myoclonic epilepsy and healthy controls.^59^ In contrast to the results in control subjects, in benign myoclonic epilepsy the N20–P25 and P25–N33 giant SEP components were potentiated by both the ‘potentiating’ (LTP-like) and ‘depressing’ [long-term depression (LTD)-like] QPS protocols.^59^ However, this differs from the present results in that the QPS was applied directly to M1 rather than to cerebellum, which only has indirect effects on M1.

A second possible explanation for our results is that in cortical myoclonus the effect of ac-tDCS differs from that in healthy controls. It is possible that Purkinje cell-DCN synapses respond oppositely to Purkinje polarization produced by ac-tDCS: synaptic effectiveness could be suppressed rather than enhanced. In the normal brain, enhanced efficacy of these inhibitory synapses reduces nuclear output leading to reduced cerebellar facilitation of cortex, whereas in cortical myoclonus reduced synaptic efficacy would enhance nuclear output and increase facilitation of M1. Although it would be very unlikely that any pathophysiology could reverse the response of Purkinje cells to hyperpolarization and depolarization by tDCS, it is important to remember that while anodal stimulation depolarizes the cell body, it hyperpolarizes the dendrites in animals (non-mammalian).^60,61^ Predicting the responses of Purkinje cells in the human cerebellum *in vivo* is difficult,^61^ but, if similar mechanisms occur, dendritic hyperpolarization might reduce the parallel fibre input that drives the rate of simple spike discharge and lower the Purkinje cells discharge. In patients affected by cortical myoclonus, there is pathological evidence of cerebellar degeneration, with sparing of the dentate and significant Purkinje cell loss symmetrically involving all lobules of the cerebellum.^15^ Whether the severe Purkinje cell loss is implicated in the reduced inhibition to the dentate nuclei and ipsilateral motor cortex or responsible of the abnormal response to tDCS is difficult to demonstrate *in vivo*, but interesting to explore.

### Anodal cerebellar transcranial direct current stimulation effect on myoclonus

Although it did not reach statistical significance, inspection of the EMG records showed that there was a trend towards deterioration of the myoclonus after cerebellar tDCS, which would be consistent with the increase in cortical excitability as reflected in the SEP and LLR-I. However, evidence suggests that there may not be a direct relationship between sensorimotor cortical excitability and the severity of cortical myoclonus. For instance, a previous study found that in the untreated state, the size of P25 and N33 components of the enlarged SEP were correlated with EMG of the jerks, but this could be dissociated by the intravenous administration of lisuride or clonazepam, which reduced the severity of the myoclonic jerks but had no effect, or even increased, the amplitude of the SEPs.^38^ Two other studies showed improvement of the myoclonus and reduction of the SEPs amplitude after intravenous injection of 5-hydroxytryptophan and perampanel,^62,63^ but without any correlation between the changes in SEP amplitudes and the clinical myoclonus scores.^62^ Thus, although there may be no direct relationship between the degree of cortex excitability (as shown at least by SEP amplitude) and severity of the jerks, our findings suggest that the reduced sensorimotor inhibition induced by the cerebellar stimulation might negatively affect myoclonus, which could be an interesting avenue for new forms of treatment or treatment targets for cortical myoclonus.

No parallel changes were found in the UMRS after ac-tDCS, very likely because the clinical scale is not sensitive enough to detect the increase of EMG activity observed after the stimulation. We cannot exclude a possible placebo effect of ac-tDCS on the severity of myoclonus, assessed by recording of continuous EMG activity, as it is known that involuntary movements may be affected by a large number of variables.^64^ However, this phenomenon would not be obvious in the present case, as patients were not informed about possible improvement or worsening of the myoclonus due to experimental procedure. The only information conveyed was our intent to explore the role of the cerebellum on several electrophysiological measures.

### Limitations and conclusion

Some limitations of the study should be addressed. First, our sample of patients is clinically heterogeneous, as the patients are affected by different cortical myoclonus syndromes. However, the variables considered are all related to the presence of cortical myoclonus and not strictly dependent on the pathophysiology underlying the condition. This is valid not only for SEP and LLR, but also for SICI as it is normally found as reduced in cortical myoclonus syndromes and indicative of reduced motor inhibition. Second, the sample is small, but it reflects the rarity of this condition and difficulty of studying these patients, which are often also severely affected by other symptoms. As cathodal tDCS was not applied, we cannot exclude that the unexpected facilitation of sensorimotor excitability was due to a defective polarity-specific tDCS effect. The general rule of anodal being excitatory and cathodal inhibitory is probably an oversimplification of the physiological mechanisms underlying tDCS, as numerous factors can turn facilitatory changes into inhibitory, and vice versa.^21^ However, although we did not measure cerebellar-brain inhibition to prove our hypothesis, many studies have demonstrated that anodal tDCS increases the inhibitory action of the cerebellum to M1,^23-26^ while the dual tDCS effect over the cerebellum has also been confirmed by behavioural studies.^52^ We do not believe that the paradoxical response could be attributed to cerebellar atrophy because only two patients had reduced cerebellar volume and these patients’ results were in line with the trend of the whole group. Moreover, in previous studies on patients with cerebellar ataxia and cerebellar atrophy, ac-tDCS was able to improve the symptoms as well as restore cerebellar-brain inhibition,^25,26^ indicating that cerebellar atrophy does not restrain the ac-tDCS effect. Finally, we acknowledge that drug washout could not be complete for certain medications, and we cannot exclude that this might have influenced the results; however, we believe that this does not account for the post ac-tDCS effect, since it was performed 1–1.5 h after the baseline assessment and it is very unlikely that the drug concentration in the blood changed in this short period of time to a degree that could have affected the post-tDCS responses.

In conclusion, our data showed that ac-tDCS facilitates, and does not inhibit, sensorimotor cortex excitability in cortical myoclonus syndromes. This paradoxical response might be due to an abnormal homeostatic plasticity within the sensorimotor cortex, likely driven by a dysfunction of the cerebellar input to the motor cortex, via CTC projection. The data also provide further evidence that the cerebellum is implicated in the pathophysiology of cortical myoclonus and could open the way to new forms of treatment or treatment targets for cortical myoclonus.

## Data Availability

The data that support the findings of this study are available from the corresponding author, upon reasonable request.

## References

[1] Marsden CD , Hallett M, Fahn S. The nosology and pathophysiology of myoclonus. In: Marsden CD, Fahn S, eds. Movement Disorders. Butterworth-Heinemann; 1981:196–248.

[2] Latorre A , Rocchi L, Berardelli A, Rothwell JC, Bhatia KP, Cordivari C. Reappraisal of cortical myoclonus: A retrospective study of clinical neurophysiology. Mov Disord. 2018;33:339–341. 29193325 10.1002/mds.27234

[3] Shibasaki H , Hallett M. Electrophysiological studies of myoclonus. Muscle Nerve. 2005;31:157–174. 15547927 10.1002/mus.20234

[4] Caviness JN . Pathophysiology and treatment of myoclonus. Neurol Clin. 2009;27:757–77, vii. 19555830 10.1016/j.ncl.2009.04.002

[5] Nardone R , Versace V, Holler Y, et al Transcranial magnetic stimulation in myoclonus of different aetiologies. Brain Res Bull. 2018;140:258–269. 29803873 10.1016/j.brainresbull.2018.05.016

[6] Dubbioso R , Striano P, Tomasevic L, et al Abnormal sensorimotor cortex and thalamo-cortical networks in familial adult myoclonic epilepsy type 2: Pathophysiology and diagnostic implications. Brain Commun. 2022;4:fcac037. 10.1093/braincomms/fcac037 PMC8882005 35233526

[7] Hanajima R , Okabe S, Terao Y, et al Difference in intracortical inhibition of the motor cortex between cortical myoclonus and focal hand dystonia. Clin Neurophysiol. 2008;119:1400–1407. 18387338 10.1016/j.clinph.2008.02.009

[8] Hanajima R , Ugawa Y, Terao Y, Ogata K, Kanazawa I. Ipsilateral cortico-cortical inhibition of the motor cortex in various neurological disorders. J Neurol Sci. 1996;140(1–2):109–116. 8866435 10.1016/0022-510x(96)00100-1

[9] Brown P , Ridding MC, Werhahn KJ, Rothwell JC, Marsden CD. Abnormalities of the balance between inhibition and excitation in the motor cortex of patients with cortical myoclonus. Brain. 1996;119(Pt 1):309–317. 8624691 10.1093/brain/119.1.309

[10] Latorre A , Rocchi L, Magrinelli F, et al Unravelling the enigma of cortical tremor and other forms of cortical myoclonus. Brain. 2020;143:2653–2663. 32417917 10.1093/brain/awaa129

[11] Obeso JA , Rothwell JC, Marsden CD. The spectrum of cortical myoclonus. From focal reflex jerks to spontaneous motor epilepsy. Brain. 1985;108(Pt 1):193–124. 3919883 10.1093/brain/108.1.193

[12] Rocchi L , Latorre A, Ibanez Pereda J, et al A case of congenital hypoplasia of the left cerebellar hemisphere and ipsilateral cortical myoclonus. Mov Disord. 2019;34:1745–1747. 31609490 10.1002/mds.27881

[13] Ganos C , Kassavetis P, Erro R, Edwards MJ, Rothwell J, Bhatia KP. The role of the cerebellum in the pathogenesis of cortical myoclonus. Mov Disord. 2014;29:437–443. 24634361 10.1002/mds.25867

[14] Bhatia KP , Brown P, Gregory R, et al Progressive myoclonic ataxia associated with coeliac disease. The myoclonus is of cortical origin, but the pathology is in the cerebellum. Brain. 1995;118(Pt 5):1087–1093. 7496772 10.1093/brain/118.5.1087

[15] Tijssen MA , Thom M, Ellison DW, et al Cortical myoclonus and cerebellar pathology. Neurology. 2000;54:1350–1356. 10746609 10.1212/wnl.54.6.1350

[16] Priori A . Brain polarization in humans: A reappraisal of an old tool for prolonged non-invasive modulation of brain excitability. Clin Neurophysiol. 2003;114:589–595. 12686266 10.1016/s1388-2457(02)00437-6

[17] Rocchi L , Spampinato DA, Pezzopane V, et al Cerebellar noninvasive neuromodulation influences the reactivity of the contralateral primary motor cortex and surrounding areas: A TMS-EMG-EEG study. Cerebellum. 2023;22:319–331. 35355218 10.1007/s12311-022-01398-0

[18] Woods AJ , Antal A, Bikson M, et al A technical guide to tDCS, and related non-invasive brain stimulation tools. Clin Neurophysiol. 2016;127:1031–1048. 26652115 10.1016/j.clinph.2015.11.012 PMC4747791

[19] Priori A , Ciocca M, Parazzini M, Vergari M, Ferrucci R. Transcranial cerebellar direct current stimulation and transcutaneous spinal cord direct current stimulation as innovative tools for neuroscientists. J Physiol. 2014;592:3345–3369. 24907311 10.1113/jphysiol.2013.270280 PMC4229333

[20] Liu A , Voroslakos M, Kronberg G, et al Immediate neurophysiological effects of transcranial electrical stimulation. Nat Commun. 2018;9:5092. 30504921 10.1038/s41467-018-07233-7 PMC6269428

[21] Grimaldi G , Argyropoulos GP, Bastian A, et al Cerebellar transcranial direct current stimulation (ctDCS): A novel approach to understanding cerebellar function in health and disease. Neuroscientist. 2016;22:83–97. 25406224 10.1177/1073858414559409 PMC4712385

[22] Galea JM , Jayaram G, Ajagbe L, Celnik P. Modulation of cerebellar excitability by polarity-specific noninvasive direct current stimulation. J Neurosci. 2009;29:9115–9122. 19605648 10.1523/JNEUROSCI.2184-09.2009 PMC2760225

[23] Naeije G , Rovai A, Destrebecq V, Trotta N, De Tiège X. Anodal cerebellar transcranial direct current stimulation reduces motor and cognitive symptoms in Friedreich's ataxia: A randomized, sham-controlled trial. Mov Disord. 2023;38:1443–1450. 37310043 10.1002/mds.29453

[24] Takano K , Katagiri N, Sato T, et al Changes in corticospinal excitability and motor control during cerebellar transcranial direct current stimulation in healthy individuals. Cerebellum. 2022;22:905–914. 36053392 10.1007/s12311-022-01469-2

[25] Benussi A , Cantoni V, Manes M, et al Motor and cognitive outcomes of cerebello-spinal stimulation in neurodegenerative ataxia. Brain. 2021;144:2310–2321. 33950222 10.1093/brain/awab157

[26] Benussi A , Dell'Era V, Cotelli MS, et al Long term clinical and neurophysiological effects of cerebellar transcranial direct current stimulation in patients with neurodegenerative ataxia. Brain Stimul. 2017;10:242–250. 27838276 10.1016/j.brs.2016.11.001

[27] Latorre A , Hale B, Rocchi L. How do I find clues about where myoclonus is originating? Mov Disord Clin Pract. 2022;9:721–722. 35844279 10.1002/mdc3.13472 PMC9274365

[28] Cruccu G , Aminoff MJ, Curio G, et al Recommendations for the clinical use of somatosensory-evoked potentials. Clin Neurophysiol. 2008;119:1705–1719. 18486546 10.1016/j.clinph.2008.03.016

[29] Rocchi L , Erro R, Antelmi E, et al High frequency somatosensory stimulation increases sensori-motor inhibition and leads to perceptual improvement in healthy subjects. Clin Neurophysiol. 2017;128:1015–1025. 28463818 10.1016/j.clinph.2017.03.046

[30] Anzellotti F , Onofrj M, Bonanni L, Saracino A, Franciotti R. Giant early components of somatosensory evoked potentials to tibial nerve stimulation in cortical myoclonus. Neuroimage Clin. 2016;12:212–218. 27489768 10.1016/j.nicl.2016.07.001 PMC4949734

[31] Visani E , Canafoglia L, Rossi Sebastiano D, et al Giant SEPs and SEP-recovery function in Unverricht-Lundborg disease. Clin Neurophysiol. 2013;124:1013–1018. 23276489 10.1016/j.clinph.2012.11.011

[32] Vidal-Dourado M , Nunes KF, Guaranha MS, Giuliano LM, Yacubian EM, Manzano GM. Expression of praxis induction on cortical excitability in juvenile myoclonic epilepsy. Clin Neurophysiol. 2016;127:2551–2560. 27291873 10.1016/j.clinph.2016.03.028

[33] Deuschl G , Eisen A. Long-latency reflexes following electrical nerve stimulation. The Inter national Federation of Clinical Neurophysiology. Electroencephalogr Clin Neurophysiol Suppl. 1999;52:263–268. 10590995

[34] Cruccu G , Deuschl G. The clinical use of brainstem reflexes and hand-muscle reflexes. Clin Neurophysiol. 2000;111:371–387. 10699396 10.1016/s1388-2457(99)00291-6

[35] Rossini PM , Barker AT, Berardelli A, et al Non-invasive electrical and magnetic stimulation of the brain, spinal cord and roots: Basic principles and procedures for routine clinical application. Report of an IFCN committee. Electroencephalogr Clin Neurophysiol. 1994;91:79–92. 7519144 10.1016/0013-4694(94)90029-9

[36] Kujirai T , Caramia MD, Rothwell JC, et al Corticocortical inhibition in human motor cortex. J Physiol. 1993;471:501–519. 8120818 10.1113/jphysiol.1993.sp019912 PMC1143973

[37] Lefaucheur JP , Antal A, Ayache SS, et al Evidence-based guidelines on the therapeutic use of transcranial direct current stimulation (tDCS). Clin Neurophysiol. 2017;128:56–92. 27866120 10.1016/j.clinph.2016.10.087

[38] Rothwell JC , Obeso JA, Marsden CD. On the significance of giant somatosensory evoked potentials in cortical myoclonus. J Neurol Neurosurg Psychiatry. 1984;47:33–42. 6420519 10.1136/jnnp.47.1.33 PMC1027638

[39] Zutt R , Elting JW, van Zijl JC, et al Electrophysiologic testing aids diagnosis and subtyping of myoclonus. Neurology. 2018;90:e647–e657. 29352095 10.1212/WNL.0000000000004996 PMC5818165

[40] Insola A , Di Lazzaro V, Assenza G. Cortical inhibitory dysfunction in epilepsia partialis continua: A high frequency oscillation somatosensory evoked potential study. Clin Neurophysiol. 2019;130:439–444. 30769270 10.1016/j.clinph.2019.01.005

[41] Sutton GG , Mayer RF. Focal reflex myoclonus. J Neurol Neurosurg Psychiatry. 1974;37:207–217. 4819909 10.1136/jnnp.37.2.207 PMC494614

[42] Kurtzer IL . Long-latency reflexes account for limb biomechanics through several supraspinal pathways. Front Integr Neurosci. 2015;8:99. 25688187 10.3389/fnint.2014.00099 PMC4310276

[43] Fong PY , Spampinato D, Rocchi L, et al Two forms of short-interval intracortical inhibition in human motor cortex. Brain Stimul. 2021;14:1340–1352. 34481097 10.1016/j.brs.2021.08.022 PMC8460995

[44] Hannah R , Rocchi L, Tremblay S, Wilson E, Rothwell JC. Pulse width biases the balance of excitation and inhibition recruited by transcranial magnetic stimulation. Brain Stimul. 2020;13:536–538. 32289672 10.1016/j.brs.2020.01.011

[45] Hamada M , Strigaro G, Murase N, et al Cerebellar modulation of human associative plasticity. J Physiol. 2012;590:2365–2374. 22473780 10.1113/jphysiol.2012.230540 PMC3424758

[46] Restuccia D , Valeriani M, Barba C, et al Functional changes of the primary somatosensory cortex in patients with unilateral cerebellar lesions. Brain. 2001;124(Pt 4):757–768. 11287375 10.1093/brain/124.4.757

[47] Grimaldi G , Manto M. Anodal transcranial direct current stimulation (tDCS) decreases the amplitudes of long-latency stretch reflexes in cerebellar ataxia. Ann Biomed Eng. 2013;41:2437–2447. 23780473 10.1007/s10439-013-0846-y

[48] Manto M . Mechanisms of human cerebellar dysmetria: Experimental evidence and current conceptual bases. J Neuroeng Rehabil. 2009;6:10. 19364396 10.1186/1743-0003-6-10 PMC2679756

[49] Ates MP , Alaydin HC, Cengiz B. The effect of the anodal transcranial direct current stimulation over the cerebellum on the motor cortex excitability. Brain Res Bull. 2018;140:114–119. 29704512 10.1016/j.brainresbull.2018.04.012

[50] Diener HC , Dichgans J, Bacher M, Guschlbauer B. Characteristic alterations of long-loop “reflexes” in patients with Friedreich's disease and late atrophy of the cerebellar anterior lobe. J Neurol Neurosurg Psychiatry. 1984;47:679–685. 6747644 10.1136/jnnp.47.7.679 PMC1027894

[51] Allen GI , Tsukahara N. Cerebrocerebellar communication systems. Physiol Rev. 1974;54:957–1006. 4370744 10.1152/physrev.1974.54.4.957

[52] Jayaram G , Tang B, Pallegadda R, Vasudevan EV, Celnik P, Bastian A. Modulating locomotor adaptation with cerebellar stimulation. J Neurophysiol. 2012;107:2950–2957. 22378177 10.1152/jn.00645.2011 PMC3378372

[53] Kronberg G , Bridi M, Abel T, Bikson M, Parra LC. Direct current stimulation modulates LTP and LTD: Activity dependence and dendritic effects. Brain Stimul. 2017;10:51–58. 28104085 10.1016/j.brs.2016.10.001 PMC5260488

[54] Khedr EM , Gilio F, Rothwell J. Effects of low frequency and low intensity repetitive paired pulse stimulation of the primary motor cortex. Clin Neurophysiol. 2004;115:1259–1263. 15134692 10.1016/j.clinph.2003.08.025

[55] Münchau A , Bloem BR, Irlbacher K, Trimble MR, Rothwell JC. Functional connectivity of human premotor and motor cortex explored with repetitive transcranial magnetic stimulation. J Neurosci. 2002;22:554–561. 11784802 10.1523/JNEUROSCI.22-02-00554.2002 PMC6758651

[56] Houdayer E , Devanne H, Tyvaert L, Defebvre L, Derambure P, Cassim F. Low frequency repetitive transcranial magnetic stimulation over premotor cortex can improve cortical tremor. Clin Neurophysiol. 2007;118:1557–1562. 17531531 10.1016/j.clinph.2007.04.014

[57] Rossi Sebastiano D , Cazzato D, Visani E, et al Significance and clinical suggestions for the somatosensory evoked potentials increased in amplitude revealed by a large sample of neurological patients. Neurol Sci. 2022;43:5553–5562. 35759065 10.1007/s10072-022-06236-z

[58] Karabanov A , Ziemann U, Hamada M, et al Consensus paper: Probing homeostatic plasticity of human Cortex with non-invasive transcranial brain stimulation. Brain Stimul. 2015;8:993–1006. 26598772 10.1016/j.brs.2015.06.017

[59] Nakatani-Enomoto S , Hanajima R, Hamada M, et al Somatosensory-evoked potential modulation by quadripulse transcranial magnetic stimulation in patients with benign myoclonus epilepsy. Clin Neurophysiol. 2016;127:1560–1567. 26431618 10.1016/j.clinph.2015.07.029

[60] Chan CY , Hounsgaard J, Nicholson C. Effects of electric fields on transmembrane potential and excitability of turtle cerebellar Purkinje cells in vitro. J Physiol. 1988;402:751–771. 3236254 10.1113/jphysiol.1988.sp017232 PMC1191919

[61] Asan AS , Lang EJ, Sahin M. Entrainment of cerebellar Purkinje cells with directional AC electric fields in anesthetized rats. Brain Stimul. 2020;13:1548–1558. 32919090 10.1016/j.brs.2020.08.017 PMC7722055

[62] Oi K , Neshige S, Hitomi T, et al Low-dose perampanel improves refractory cortical myoclonus by the dispersed and suppressed paroxysmal depolarization shifts in the sensorimotor cortex. Clin Neurophysiol. 2019;130:1804–1812. 31401489 10.1016/j.clinph.2019.07.006

[63] Shibasaki H , Yamashita Y, Neshige R, Tobimatsu S, Fukui R. Pathogenesis of giant somatosensory evoked potentials in progressive myoclonic epilepsy. Brain. 1985;108(Pt 1):225–240. 3919884 10.1093/brain/108.1.225

[64] Berlot R , Rothwell JC, Bhatia KP, Kojović M. Variability of movement disorders: The influence of sensation, action, cognition, and emotions. Mov Disord. 2021;36:581–593. 33332680 10.1002/mds.28415

